# Progress in mimicking brain microenvironments to understand and treat neurological disorders

**DOI:** 10.1063/5.0043338

**Published:** 2021-04-08

**Authors:** Mai T. Ngo, Brendan A. C. Harley

**Affiliations:** 1 Department of Chemical and Biomolecular Engineering, University of Illinois at Urbana-Champaign, Urbana, Illinois 61801, USA; 2 Carl R. Woese Institute for Genomic Biology, University of Illinois at Urbana-Champaign, Urbana, Illinois 61801, USA; 3 Cancer Center at Illinois, University of Illinois at Urbana-Champaign, Urbana, Illinois 61801, USA

## Abstract

Neurological disorders including traumatic brain injury, stroke, primary and metastatic brain tumors, and neurodegenerative diseases affect millions of people worldwide. Disease progression is accompanied by changes in the brain microenvironment, but how these shifts in biochemical, biophysical, and cellular properties contribute to repair outcomes or continued degeneration is largely unknown. Tissue engineering approaches can be used to develop *in vitro* models to understand how the brain microenvironment contributes to pathophysiological processes linked to neurological disorders and may also offer constructs that promote healing and regeneration *in vivo*. In this Perspective, we summarize features of the brain microenvironment in normal and pathophysiological states and highlight strategies to mimic this environment to model disease, investigate neural stem cell biology, and promote regenerative healing. We discuss current limitations and resulting opportunities to develop tissue engineering tools that more faithfully recapitulate the aspects of the brain microenvironment for both *in vitro* and *in vivo* applications.

## I. INTRODUCTION

Neurological disorders, which encompass traumatic brain injuries, cerebrovascular injuries such as stroke, brain tumors, and neurodegenerative diseases, affect millions of people worldwide. For example, there were roughly 70 × 10^6^ cases of traumatic brain injury (TBI) annually, 80 × 10^6^ cases of stroke worldwide, and at least 300 000 cases of central nervous system cancers worldwide in 2016.^1–3^ In the United States, stroke and Alzheimer's disease are among the top ten leading causes of death.^4^ The societal burden of these disorders is expected to increase due to their prevalence among the elderly, which is a population that continues to grow due to prolonged life expectancy. The often incurable nature of these disorders underscores a critical need to develop novel experimental tools as disease models, as well as therapeutic strategies to halt disease progression or enhance regenerative outcomes. However, beyond neurological disorders, there is also broad interest in developing models of the brain for applications such as toxicology screening, fundamental neuroscience, and even artificial intelligence.

The microenvironment of a tissue is known to influence its function, and the brain is no exception.^5^ Biophysical, biochemical, and cellular cues coordinate to maintain normal brain activity during homeostasis, promote repair or regeneration after an insult, and can be drastically altered during pathophysiological conditions. While numerous studies have detailed how the brain microenvironment is altered during injury or disease, it is largely unknown how these changes might facilitate or impede repair.^6–8^ Yet, growing knowledge of the role of the brain microenvironment in homeostasis and regeneration has inspired tissue engineering strategies to bolster outcomes in disorders such as traumatic brain injury, stroke, and multiple sclerosis.^9–11^ These design principles have been further applied to generate models for cancer and neurodegenerative diseases,^12,13^ and mechanistic insights from these platforms will eventually lead to strategies to slow and potentially cure these disorders.

In this Perspective, we provide an overview of the biophysical, biochemical, and cellular components of the brain microenvironment during both normal and pathophysiological states. We then discuss the recent developments in engineered platforms that recapitulate aspects of the brain microenvironment for *in vitro* studies and *in vivo* regeneration. We place particular emphasis on recent innovations to model neurodegenerative disease and develop brain-on-chip platforms. Throughout, we will highlight the opportunities for novel technologies to further enhance the ability to mimic the brain microenvironment for applications ranging from fundamental biology to clinical translation.

## II. COMPONENTS OF THE BRAIN MICROENVIRONMENT

### A. Biophysical properties

Cells sense the mechanical properties of the surrounding microenvironment through cell–matrix contacts mediated by integrins, cell–cell contacts via cadherins, and stretch-activated ion channels, among others.^14–17^ Mechanotransduction pathways enable mechanical stimuli to instruct cell behavior and influence migration, proliferation, extracellular matrix (ECM) remodeling, and stem cell phenotype.^18–20^ Within the developing brain, for example, durotaxis mediated by the stretch-activated ion channel piezo1 directs axon pathfinding.^21^ Furthermore, tensile strain sensed through integrin α6 has been shown to affect the ability of neural stem cells (NSCs) to differentiate into oligodendrocytes.^22^ Work from David Schaffer, Sanjay Kumar, and colleagues also demonstrates that NSC proliferation and differentiation is also mechanosensitive through pathways such as yes-associated protein 1 (YAP) and Rho GTPase.^23–25^ Increasing evidence directly ties mechanosensing with brain tumor phenotype, in which matrix stiffness has been shown to influence proliferation, migration, and cell spreading in glioblastoma (GBM), the most common and lethal form of primary brain cancer.^26^ Increased tissue stiffness has been correlated with tumor grade and aggression,^27^ and several positive feedback systems that increase tissue stiffness and mechanosensing have been identified, including overexpression of Piezo1, crosslinking enzyme lysyl oxidase, and guidance receptors such as plexins.^28–30^ Beyond stiffness, GBM cells also respond to a class of mechanical signals initiated by interstitial flow, which increases invasion through CXCL12-CXCR4 and CD44 signaling.^31^

The brain is a viscoelastic material, and its mechanical properties have been measured by a range of indentation techniques and magnetic resonance elastography.^14,32,33^ For adult mammalian tissue, Young's and storage moduli range between 1 and 5 kPa,^33–35^ while Sack *et al.* report shear viscosity and loss modulus values of 3 Pa s and 0.5 kPa, respectively.^35,36^ Additionally, the mechanical properties of the brain vary with spatial location, sex, and age. Elkin *et al.* used atomic force microscopy (AFM) to investigate the mechanical properties of rat brains, reporting increases in elastic modulus from ∼0.1 kPa to ∼1 kPa during post-natal development to adulthood.^33^ In adult samples, both Arani *et al.* and Sack *et al.* report a decrease in tissue stiffness with age, although Arani *et al.* note that no correlation existed in the cerebellum and sensory motor regions of the brain.^35,37^ Furthermore, some evidence suggests sex-based differences, with female brain tissue being stiffer than the male counterpart.^35,37^ Female brain tissue is approximately 100 Pa stiffer than male tissue, suggesting that the female brain is “younger” than the male counterpart in age-matched individuals. Budday *et al.* and Weickenmeier *et al.* also report differences in elastic modulus between white and gray matter, with white matter being stiffer.^38,39^

The mechanical microenvironment within the brain remodels dynamically during the onset and progression of pathophysiological conditions. For brain tumors, Miroshnikova *et al.* used AFM methods to demonstrate that tissue stiffness increases with tumor grade, and recurring tumors additionally are as stiff or stiffer than primary tumors.^27^ There is also a dynamic relationship between increased stiffness, deposition of tenascin C (TNC) protein, and decreased survival. On the other hand, neurodegenerative disorders, traumatic brain injury, and stroke are often accompanied by decreased tissue stiffness.^40–45^ However, the implications of altered tissue mechanics on injury or disease progression are not well-understood, making the development of tools to investigate the influence of matrix remodeling on cell phenotype an important ongoing area of research.

In addition to tissue viscoelasticity, another mechanical input that affects cell phenotype is shear stress.^46^ While shear stress has largely been explored in the context of endothelial cell biology and vascular fluid flow, Munson *et al.* recently demonstrated the use of methods such as magnetic resonance imaging to generate velocity maps of interstitial fluid flow within the brain in order to demonstrate that interstitial fluid flow is sensed by GBM cells and can enhance their invasive capacity.^47–49^ Recent work by Householder *et al.* also suggested the use of interstitial flow as a potential delivery agent of nanomedicines within the central nervous system.^50^ As a result, abundant opportunities remain to explore the role of *in situ* imaging to define the status of interstitial fluid flow in neural stem cell biology, regeneration, and neurodegenerative disorders, as well as to explore the use of interstitial flow as a delivery mechanism to boost healing or combat pathologies.

### B. Biochemical properties

**FIG. 1.**
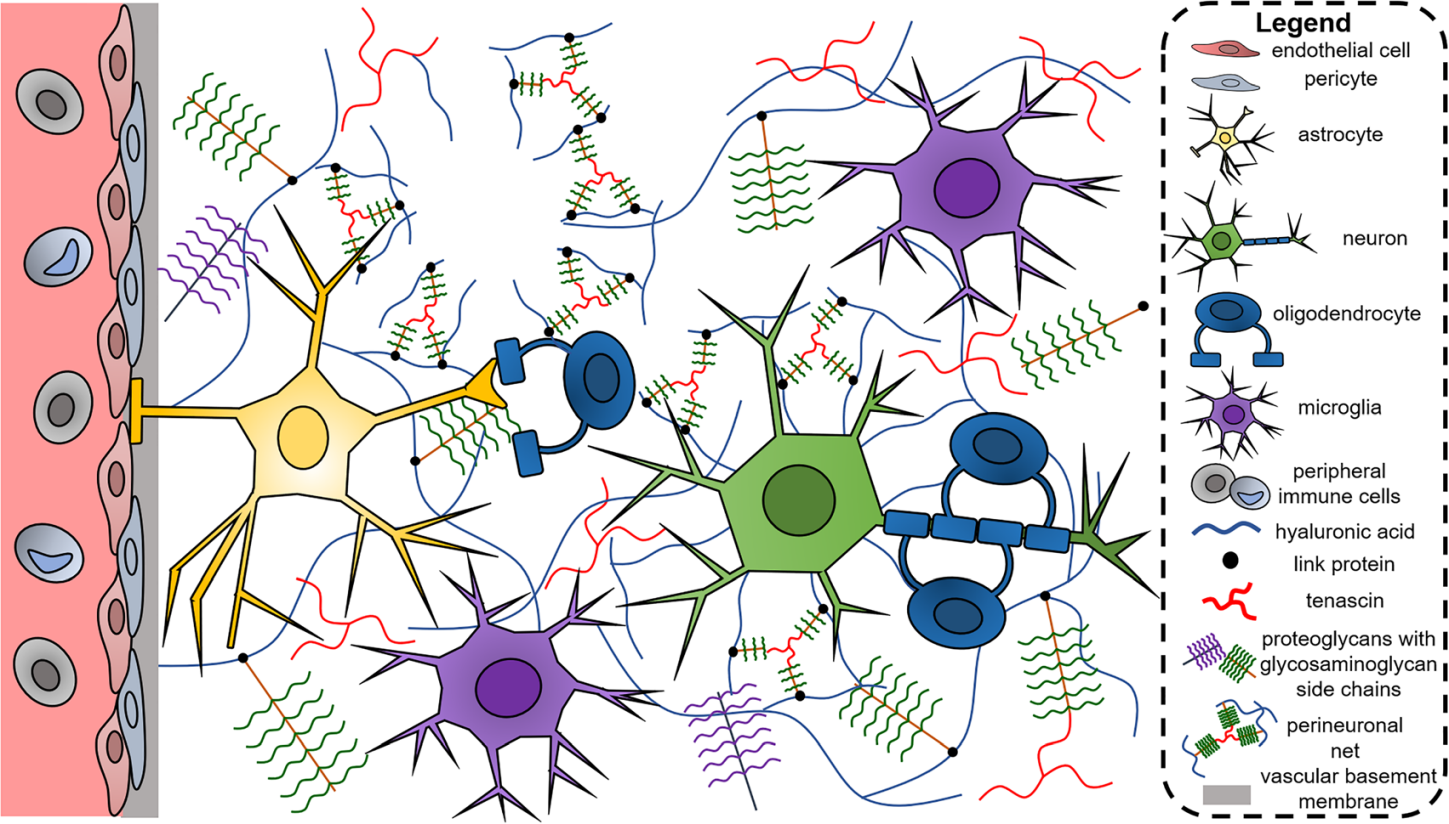
The brain extracellular matrix is largely composed of non-fibrillar components such as hyaluronic acid, proteoglycans, glycosaminoglycans, and tenascins. Collagens are largely confined to the vascular basement membrane along with proteins, such as fibronectin and laminin, but increased expression and deposition of these proteins are observed in neurological disorders (e.g., brain tumors). Hyaluronic acid can bind to chondroitin sulfate proteoglycans via link proteins, and along with tenascins form perineuronal nets on the surfaces of neurons. Besides neurons, cell types in the brain include vascular cells (e.g., endothelial cells and pericytes), glial cells (e.g., astrocytes, oligodendrocytes, microglia), and immune cells (e.g., microglia). Peripheral immune cells are largely confined to vasculature, but in events where the blood–brain barrier is compromised, these cells can enter the brain parenchyma and contribute to neuroinflammation.

In contrast to other tissues that contain an abundance of fibrous collagen, the extracellular matrix (ECM) of the brain during homeostasis is largely non-fibrillar and instead contains proteoglycans (PGs), glycosaminoglycans (GAGs), and glycoproteins such as tenascins (Fig. 1).^51,52^ Matrix proteins can be randomly dispersed throughout the ECM or organized in perineuronal nets (PNNs), which are ECM meshes deposited on the surfaces of neurons. PNNs are composed of PGs, tenascins, and hyaluronic acid (HA), and contribute to neuronal function and plasticity.^53^ Collagens are present in the vascular basement membrane along with fibronectin and laminin.^6^ Here, we will further discuss the influence of matrix proteins (PGs and GAGs, tenascins, and basement membrane) in normal brain function and disease.

#### 1. Proteoglycans and glycosaminoglycans

GAGs that are present in the brain include chondroitin sulfate (CS), heparan sulfate (HS), and hyaluronic acid (HA).^51^ CS and HS are found as side chains of core PGs, with lecticans being one class of CS-binding PGs while glypicans and syndecans bind heparan sulfate.^54^ In contrast, HA does not directly bind to PGs, but link proteins are able to connect HA to lecticans.^55^ PGs and GAGs are instrumental in regulating growth factor binding and sequestration in the ECM, thereby controlling the spatial distribution of growth factors and their activity.^56,57^ Heparan sulfate is crucial for brain development in an embryonic mouse model, and also directs axon guidance.^58^ Heparan sulfate exerts its impact on development by ensuring proper patterning of basic fibroblast growth factor and promoting neuronal proliferation, and it has been exploited to promote *in vitro* differentiation of embryonic stem cells to neural stem cells.^59^ Increased heparan sulfate deposition is observed in Alzheimer's patients, but its role in disease progression is not well understood.^60^ Chondroitin sulfate supports neural stem cell self-renewal and proliferation and promotes differentiation into neurons from radial glial cells.^61^ Chondroitin sulfate potentially exerts its pro-proliferative effect by activating epidermal growth factor receptor (EGFR) signaling.^62^ Interestingly, the sulfation pattern of chondroitin sulfate moderates its function, with chondroitin-4-sulfate acting as an axonal repellant while chondroitin-6-sulfate has no effect on directing axonal growth.^63^ Chondroitin sulfate inhibits neural stem cell migration as well as oligodendrocyte maturation and adhesion,^64,65^ and could therefore present a barrier to neural regeneration and remyelination. Indeed, chondroitin sulfate expression is increased in GBM and supports tumor cell migration,^66^ as well as the activation and infiltration of pro-inflammatory macrophages in multiple sclerosis.^67^ Active plaques in multiple sclerosis also have increased lectican and dermatan sulfate deposition around their edges, suggesting a potential role for these molecules in the acute phase of the disease.^68^

The abundance of hyaluronic acid in the brain is a significant motivation for studying its role in the brain, particularly in pathological conditions. Our group and others have shown that hyaluronic acid, which signals through CD44 and receptor for hyaluronan mediated motility (RHAMM) receptors, influences GBM migration, proliferation, and therapeutic response.^69–71^ The hyaluronic acid landscape additionally shifts in neurodegenerative diseases. In multiple sclerosis, there is increased deposition of the molecule, while expression of remodeling genes such as hyaluronic acid synthases and hyaluronidases is differentially altered post-stroke as well as in Alzheimer's disease.^51,72^ Expression of hyaluronic acid synthases is differentially regulated, with reduced HAS1 and increased HAS3 in a murine model of Alzheimer's, resulting in increased production of low-molecular weight (<200 kDa) hyaluronic acid.^72^ Hyaluronic acid is known to exert different functions based on its molecular weight, with low-molecular weight fragments reported to be pro-inflammatory and angiogenic while high-molecular weight molecules (>400 kDa) are anti-angiogenic, anti-inflammatory, and immunosuppressive.^73–75^ Therefore, the synthesis of new oligomers and breakdown of existing ones via hyaluronic acid synthases and hyaluronidases, respectively, might influence disease progression by altering the molecular weight distribution of hyaluronic acid in the tissue.

#### 2. Tenascins

Tenascin C (TNC) and Tenascin R (TNR) influence several aspects of cell phenotype within the brain. TNC is essential for cortical development and promotes haptotaxis of neural stem cells.^76,77^ In oligodendrocyte precursors, TNC regulates migration and increases proliferation through synergy with platelet-derived growth factor (PDGF) via integrin αvβ3.^78^ Meanwhile, different domains of TNR affect proliferation and differentiation through integrin β1 binding: the FN6–8 domain reduces proliferation of neural stem cells and skews differentiation toward astrocytes, while the epidermal growth factor like (EGFL) domain promotes neuronal differentiation.^79^ TNR additionally supports radial migration of neuroblasts and their recruitment to the olfactory bulb.^80^ As a component of PNNs, the tenascins potentially protect neurons from neurodegeneration. Murine models deficient in TNR have reduced numbers of neurons wrapped in PNNs, increased lesion size following injury, and PNNs without TNR are not neuroprotective.^81^ The tenascins also contribute to pathological conditions; in GBM, increased tenascin correlates with tumor aggression and decreased survival.^27^ TNC may contribute to neuronal death post-stroke and is also potentially involved in Alzheimer's progression by increasing the inflammatory phenotype of microglia.^82,83^ In multiple sclerosis, acute plaques do not express tenascin, but expression is regained in chronic plaques, suggesting a role for the glycoprotein in long-term disease.^84^

#### 3. Basement membrane proteins

The basement membrane proteins include laminin, fibronectin, entactin, collagen type IV, and heparan sulfate proteoglycan.^85^ While the basement membrane proteins are concentrated around vasculature, they exert a large influence on brain tissue development and function. In particular, neural stem cells have been shown to reside in perivascular niches,^86^ which motivates the involvement of basement membrane proteins in maintaining the stem-like phenotype of these cells. Here, perlecan has been shown to synergize with FGF2 to regulate neural stem cell proliferation^87^ while laminin can promote proliferation, survival, and migration.^88,89^ Laminin exerts its pro-survival effect through integrin β1, while migration is facilitated through integrin α6. It also supports differentiation into neurons and astrocytes as well as neurite elongation.^89^ In addition to regulating neural stem cell behavior, laminin produced by astrocytes supports blood–brain barrier function that may restrict T lymphocyte infiltration into the brain.^90^ Laminin is actively produced by reactive astrocytes after injury, suggesting a role in the tissue repair process.^91,92^ Both laminin and fibronectin promote neurosphere outgrowth, while fibronectin also supports neurite elongation by engaging integrin α5β1.^93,94^ After traumatic brain injury and stroke, there is increased deposition of fibronectin and laminin,^95,96^ but whether this abundance is beneficial or detrimental to repair is not well understood. Alterations in basement membrane protein expression are also observed in Alzheimer's and Down syndrome, with increases in laminin deposition and mRNA synthesis as well as collagen deposition surrounding microvasculature.^97,98^ In multiple sclerosis, increased fibronectin and vitronectin correlates with microglial activation and MMP9 expression, which together may impair remyelination.^99,100^

### C. Cellular components

Broadly, the cells in the brain can be categorized as neuronal (neural stem cells and neurons), glial (astrocytes, oligodendrocytes, microglia, ependymal), vascular (endothelial cells and pericytes), and immune (myeloid, monocytes, macrophages, dendritic cells, lymphocytes) populations (Fig. 1). In the past decade, advances in single-cell technologies have revealed a truly complex constellation of cell types in the brain. Single-cell RNA-seq has been used to the map the cells in both human and murine brains, and clustering tools have allowed the identification of subcategories of cells belonging to each of the major categories listed above.^101,102^ For example, Darmanis *et al.* unveil seven neuronal subtypes within the human brain.^102^ Similar analysis has further delineated cell subtypes within vascular and immune populations.^103,104^ Furthermore, single-cell RNA-seq has been used to delineate intra- and inter-tumor cellular heterogeneity, such as transcriptomic differences between bulk and invasive tumor cells.^105,106^ Using human GBM samples, single-cell analysis reveals patient-to-patient and cell-to-cell variability in proliferation, hypoxic signature, and stemness, and that infiltrating tumor cells additionally upregulate genes related to invasion and cell survival. Furthermore, infiltrating tumor cells are accompanied by immune cells with pro-inflammatory, microglia-like characteristics, while immune cells with a macrophage phenotype and anti-inflammatory, pro-angiogenic markers are present in the tumor core. Single-cell RNA-seq is also useful for tracing the genetic origins of neurological diseases. For example, Skene *et al.* identify pyramidal cells, medium spiny neurons, and specific interneurons as carriers of genomic variants that are associated with schizophrenia.^107^

Injury and disease progression are accompanied by shifts in cell populations and activity within the brain microenvironment. After the onset of an injury or disease, the brain tissue experiences an influx of cells and proteins that contribute to an environment reminiscent of wound healing.^108^ Immune cells initially infiltrate to clear the compromised tissue of debris, and then fibroblast-like cells and endothelial progenitor cells proliferate to initiate new matrix formation for wound closure and vascular formation. Microglia and astrocytes attain a reactive phenotype and proliferate to form scars that isolate damaged, non-neuronal tissue from tissue that is still intact and functional. While the inflammatory response in the brain mostly derives from resident microglia and macrophages, damage to the blood–brain barrier in disorders such as multiple sclerosis and stroke leads to infiltration of circulating immune cells which can exacerbate tissue damage by attacking oligodendrocytes and neurons.^109,110^ Blood–brain barrier damage can also be induced by VEGF secreted by astrocytes, with the loss of cellular components such as pericytes potentially contributing to disease progression.^111,112^ Mass cytometry has been used to characterize both resident and infiltrating immune populations that accompany disease,^104,113^ and studies suggest significant changes in cell phenotypes and signaling pathways that may provide mechanistic insight into disease progression and also identify new therapeutic targets for directing the immune response toward a regenerative phenotype. Finally, stem or progenitor cells also contribute to injury or disease response by differentiating into the reactive astrocytes that form scar tissue to barricade non-functional lesions, or by differentiating into functional neurons or oligodendrocytes to aid tissue repair.^114,115^ Overall, single-cell technologies highlight the cellular heterogeneity of the brain microenvironment, and compared to bulk tissue analysis they enable identification and characterization of distinct cellular subpopulations that initiate and propagate development, regeneration, and disease. Identifying the underlying genomic, proteomic, and signaling landscapes that distinguish these subpopulations will provide new strategies for targeted next-generation therapies to facilitate tissue repair or treat disease. Furthermore, coupling single-cell workflows with computational pipelines such as trajectory inference analysis enables temporal mapping of cellular states, which allows insight into the events accompanying disease initiation and progression. Finally, single-cell technologies enable elucidation of cell–cell communication networks, thereby providing unprecedented information regarding the crosstalk between multiple cell types in the brain during homeostasis and pathophysiological events. We anticipate that single-cell workflows and analyses will continue to be an invaluable tool for unraveling and understanding the complexity of the brain microenvironment with unprecedented cellular and temporal resolution.

### D. Oxygen tension

Within the human brain, the mean oxygen pressure is between 3% and 5%, corresponding to 20–40 mm Hg.^116^ Because most cell culture studies occur in ambient air (∼20% oxygen), this calls into question the physiological relevance of results observed *in vitro* compared to *in vivo* models. Studies have demonstrated that brain-derived cells behave differently in environments with lowered oxygen pressure. Pistollato *et al.* demonstrate that neural stem cells cultured in 5% oxygen preferentially undergo expansion of neural precursor cells, while 20% oxygen instead supports differentiation into astrocytes.^117^ Hypoxia is further observed in disorders such as traumatic brain injury and GBM, with Collingridge *et al.* demonstrating that the median oxygen pressure in a group of human subjects is 5.6 mm Hg for high-grade glioma.^118,119^ GBM cells cultured in 3% oxygen have increased expression of CD133, a potential stem-cell marker, and our lab has shown that GBM cells cultured in 1% oxygen exhibit increased migration and reduced proliferation compared to 20% oxygen.^120,121^ Furthermore, hypoxia is associated with increased deposition of amyloid beta plaques, which are a well-known prognostic indicator of Alzheimer's onset and progression.^122^ These observations, along with the powerful connection between oxygen tension and larger shifts in the metabolic poise of a cell, highlight a critical need to develop improved processes to manipulate and monitor metabolic state, such as the use of oxygen-controlled incubators. Recent work from Farris *et al.* suggests approaches to actively deliver oxygen via biomaterial design,^123^ while recent work from Blatchley *et al.* and our lab have also highlighted the use of hydrogel crosslinking processes based on laccases that are inherently oxygen-consuming,^124,125^ which collectively suggests the potential to actively manipulate oxygen availability in multidimensional cultures. Cellular response to oxygen is mediated by HIF1α, which is degraded during normoxia but stabilized with lower oxygen pressure, leading to transcription of genes related to angiogenesis, apoptosis, and cell survival.^116,126^ Mice with depleted HIF-1α show stunted brain development arising from apoptotic neural cells, indicating the importance of oxygen sensing in this organ.^127^

Oxygen also affects brain tissue through its incorporation into reactive oxygen species (ROS). While moderate levels of reactive oxygen species can be beneficial toward neural stem cell self-renewal,^128^ reactive oxygen species generation is increased during disorders such as Alzheimer's, Down syndrome, and ischemia, resulting in neuronal dysfunction and apoptosis.^129–131^ Reactive oxygen species may also impair blood–brain barrier function and facilitate the infiltration of peripheral monocytes during disease.^132^ Oxidative stress also causes cell death during brain development, which reinforces the importance of controlled oxygen tension in proper tissue development and function.^133^

## III. ENGINEERED PLATFORMS

**FIG. 2.**
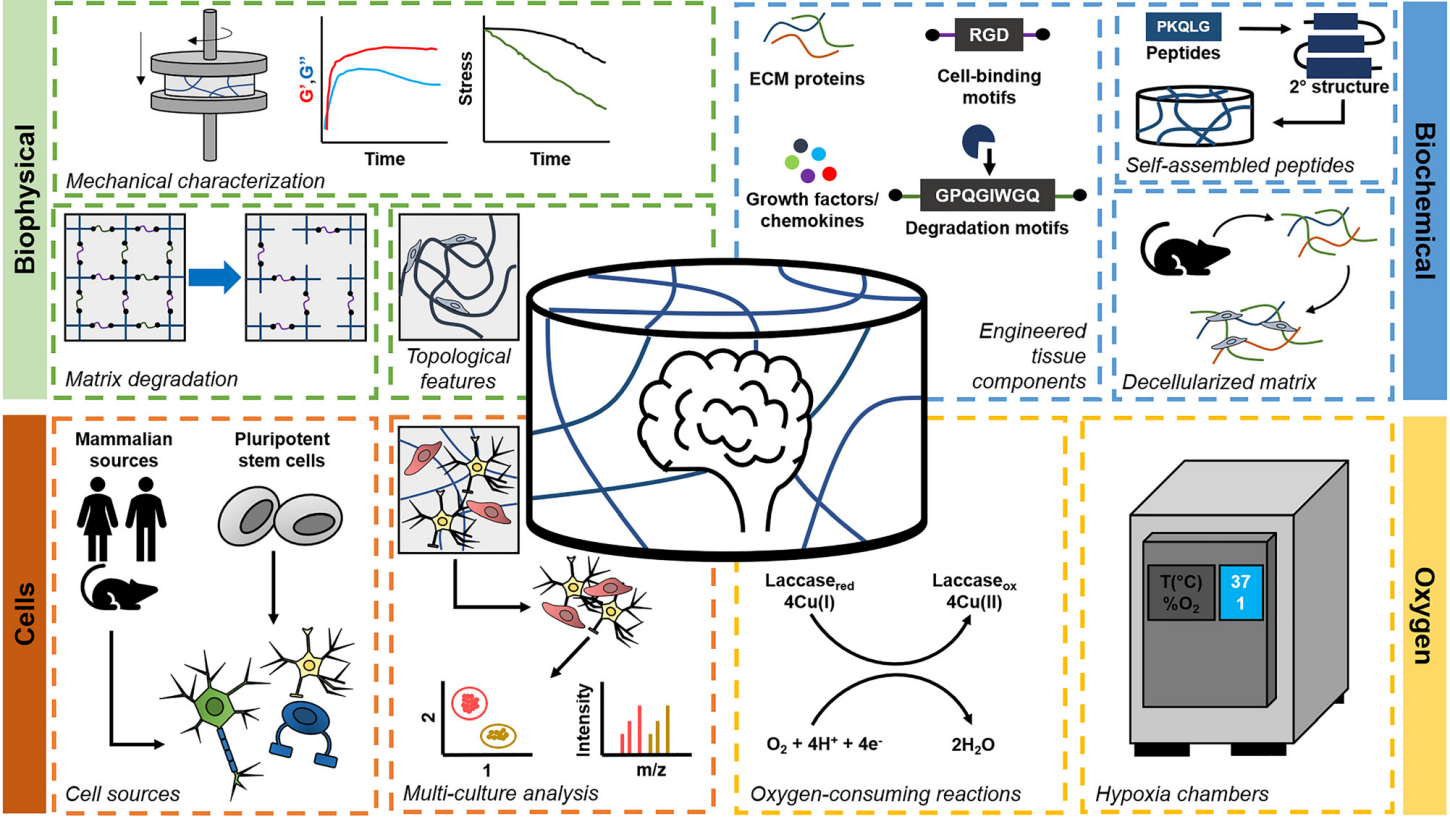
Components of engineered brain-mimetic platforms include biophysical parameters, biochemical composition, cellular composition, and oxygen content. Biophysical considerations include thorough characterization of the viscoelastic properties of the material to match native brain tissue, as well as tailoring matrix degradation and incorporating structural cues such as those mimicking white matter tracts and vasculature. Biochemical components include ECM proteins, growth factors, and peptide motifs for cells to interact with the surrounding environment. Technologies of interest include decellularized matrix and self-assembled peptide hydrogels. Cells can be obtained from primary sources or differentiated from pluripotent stem cells. Use of next-generation sequencing and chemical biology tools such as metabolic labeling will facilitate analyses of diverse cell populations. Finally, oxygen content can be controlled using chemistries that consume oxygen or by culturing platforms in hypoxia chambers.

**FIG. 3.**
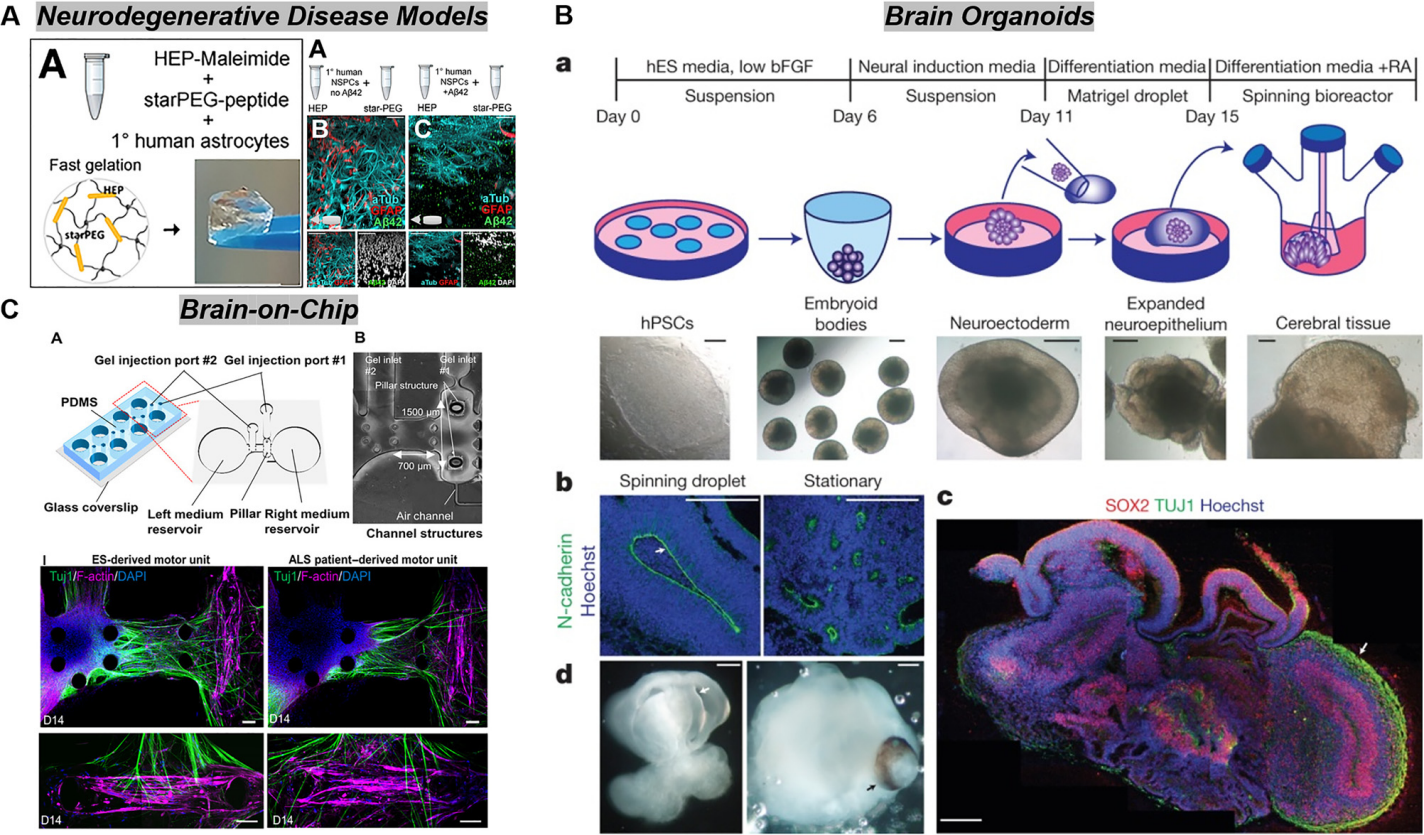
Ongoing innovations in engineering brain-mimetic platforms include the development of neurodegenerative disease models, brain organoids, and brain-on-chip platforms. (a) Neural cultures in engineered biomaterials can recapitulate several hallmarks of neurodegenerative, such as amyloid beta plaque deposition and disrupted neural networks in an Alzheimer's model.^235^ (b) Brain organoids have been developed from stem cell induction and differentiation protocols and offer great potential as models of brain development and disease. Lancaster *et al.* pioneered the self-organization of cerebral brain organoids by incorporating Matrigel encapsulation and bioreactor culture to encourage neuroepithelial commitment and proper nutrient exchange, respectively.^238^ (c) Microfluidic technologies offer advantages such as spatial organization of culture compartments, introduction of intraluminal and interstitial flow, and fluidic coupling between devices to model interactions between tissues or organs. In this figure, Osaki *et al.* generate an ALS model by culturing skeletal muscle cells and motor neurons to form a neuromuscular junction. By using neurons from an ALS patient, the neuromuscular junction is compromised compared to a healthy control.^211^ Panel (a) is reprinted with permission from Papadimitriou *et al.*, Dev. Cell **46**, 85–101 (2018). Copyright 2018 Elsevier. Panel (b) is reprinted by permission from Lancaster *et al.*, Nature **501**(7467), 373–379 (2013). Copyright 2013 Springer Nature Customer Service Centre GmbH, Springer Nature. Panel (c) is reprinted with permission from Osaki *et al.*, Sci. Adv. **4**, eaat5847 (2018). Copyright 2018 Authors, licensed under a Creative Commons Attribution (CC BY-NC) license.

In the past decade, many efforts have led to the development of engineered materials that recapitulate aspects of the brain microenvironment described above. An inherent advantage of this approach is the ability to systematically examine the role of single axes of variation on neural cell activity, with more recent efforts exploring system-level approaches to look at the role of complex cell–matrix–metabolic interactions. These platforms have been used to investigate fundamental stem cell biology, guide *in vivo* regeneration, and serve as disease models for tumors and other neurological disorders. They also motivate active and future efforts to better define the degree of complexity necessary in models of the brain microenvironment to gain critical insight about homeostasis, disease progression, and therapy. Here, we will provide a commentary on common strategies that have been utilized to recreate aspects of the biophysical, biochemical, cellular, and metabolic cues of the brain microenvironment (Fig. 2), before focusing specifically on innovations in developing neurodegenerative disease models and brain-on-chip platforms (Fig. 3).

### A. Biophysical stimuli

Engineered platforms have been used in a series of fundamental studies to elucidate the role of matrix stiffness on neural stem cell and brain tumor cell biology. In one example, Seidlits *et al.* formulated a series of hyaluronic acid hydrogels with varying degrees of methacrylate functionalization to obtain compressive moduli between 3 and 5 kPa.^134^ While materials with low modulus support neural differentiation, astrocyte differentiation is observed in materials with a high modulus. Our lab and others have investigated the role of matrix stiffness in GBM progression. 2D plating of GBM cells on hyaluronic acid matrices of increasing stiffness results in increased cell spreading, cell growth, and motility.^135^ 3D encapsulation in gelatin or hyaluronic acid hydrogels (or combinations thereof) results in increased proliferation/metabolic activity, but decreased invasion with increasing stiffness.^71,135,136^ In these studies, modulating matrix stiffness is achieved by increasing the polymer content of the hydrogel or the crosslinker concentration; however, these strategies confound matrix stiffness, density, and porosity, and the use of natural biopolymers such as collagen and gelatin additionally confounds bioactive ligand concentration. Recent work by Xiao *et al.* decouples stiffness and bioactive polymer concentration by titrating increasing amounts of bioinert polyethylene glycol (PEG) to increase stiffness while maintaining a constant amount of hyaluronic acid in a hydrogel based on PEG-maleimide.^137^ Interestingly, Wang *et al.* report reduced proliferation of GBM cells with increasing stiffness in PEG-based materials, which perhaps reinforces the notion that stiffness is confounded with ligand density and additional variables in natural-derived polymeric hydrogels.^138^ Wang *et al.* additionally report shifts in gene expression related to hyaluronic acid sensing and remodeling, matrix remodeling, *Hras*, and *Rock1* with increasing stiffness. Methods to increase hydrogel stiffness without changing architecture, such as non-enzymatic glycation,^139^ offer opportunities to decouple matrix stiffness and density but have not been applied extensively in engineered brain microenvironments.

An additional biophysical parameter of interest is matrix degradation. The degradability of a hydrogel can be tuned by incorporating varying ratios of protease-sensitive vs inert crosslinkers into synthetic hydrogels, modulating overall crosslinking density, or by varying ratios of degradable and non-degradable macromers in a material. Using the latter approach, Lampe *et al.* demonstrate that increasing matrix degradability enhances the metabolic activity and proliferation of encapsulated neural cells.^140^ In another study, hydrogels derived from elastin-like peptides are formed with different concentrations of crosslinker to tune degradability.^141^ Here, the ability of the matrix to degrade leads to maintenance of the neural stem cell population by enabling cell–cell communication and β-catenin signaling. These studies allude to matrix degradation as a potential design variable for directing neural stem cell expansion and ultimately neural regeneration *in vivo*. While materials for *in vivo* applications often include degradable sequences to facilitate neuronal and vascular infiltration, opportunities exist to further optimize the extent and timescale of degradation for ideal post-transplantation outcomes. Strategies to control degradation include mixing of crosslinker motifs with different affinities for matrix metalloproteinase activity, designing crosslinkers that can be selectively cleaved by external cues such as light, or that have varying rates for hydrolysis.^142–145^

While the brain is mostly non-fibrillar in nature, vasculature and white matter tracts form highly organized structures that serve as migratory routes for GBM cells.^146,147^ Understanding how these disparate structural cues promote GBM invasion is critical for developing new therapeutic strategies against the disease. Rao *et al.* use electrospun fibers to investigate GBM migration as a function of fiber mechanics and composition.^148^ GBM cell spreading and migration show biphasic behavior in relation to fiber stiffness: spreading and migration are maximized on fibers with an intermediate modulus of 8 MPa. When fibers are coated with different extracellular matrix proteins, GBM cells show adverse adhesion with hyaluronic acid and collagen coatings, while hyaluronic acid also has a negative effect on migration. The propensity for GBM cells to migrate along fibers has been utilized as a therapeutic strategy, in which fibers implanted *in vivo* are used to direct tumor cells toward a drug-containing collagen hydrogel.^149^ To mimic vasculature, approaches include encapsulating endothelial-laden alginate microfibers in hydrogels, which leave behind microchannels lined with endothelial cells following degradation of the alginate.^150^ Co-culture of GBM and endothelial cells in this construct results in disruption of endothelial cell–cell junctions and increased tumor cell proliferation. A related microfluidic approach described by Wolf *et al.*^151^ uses two parallel channels cast in a hyaluronic acid hydrogel, in which one channel is filled with tumor cells and the other is left empty to mimic a vascular channel. Tumor cells exhibit collective migration through the hydrogel, and upon reaching the vascular channel align and migrate along the channel wall. Collectively, these platforms demonstrate that morphological, mechanical, and biochemical signals presented by vascular walls or white matter tracts influence cell behavior. While currently utilized to understand and leverage the biophysical cues that drive tumor progression, such technologies can potentially facilitate neural stem cell migration toward injured sites post-stroke or post-traumatic brain injury.

Despite the viscoelastic nature of brain tissue, few, if any, engineered materials inspired by the brain microenvironment are fully characterized for their viscoelastic properties. Most studies report an elastic modulus derived either from compression testing or shear rheometry, while the loss modulus and time-dependent behaviors, such as stress relaxation and creep, are neglected. Calhoun *et al.* demonstrate that while a given engineered material may closely mimic brain tissue in terms of elastic properties, viscous characteristics, described in the study using a relaxation time Tau (τ), may significantly differ.^152^ Furthermore, the viscoelastic nature of materials impacts cell spreading, growth, and osteogenic and chondrogenic potential of stem cells,^153–155^ but the effects of viscoelasticity have not been explored in the context of resident brain cells. Viscoelastic properties are potentially another tunable variable to control neural stem cell fate and regeneration and may also contribute to neurological disease progression. Moving forward, we believe that it will be essential for materials to be characterized in terms of elastic properties as well as stress relaxation and creep profiles to better describe the viscoelastic nature of each multidimensional model system. Furthermore, studies should be designed to decouple elastic modulus and parameters characterizing viscous behavior (e.g., relaxation time) to elucidate the role of viscoelastic material properties in neural stem cell expansion and fate, as well as in the behavior of cells implicated in disease.^154,156^

### B. Biochemical signaling

The effect of ECM composition on cell phenotype, as well as the ability of ECM to sequester and release growth factors, underlies the motivation to create engineered materials that mimic both the biochemical signature of the ECM as well as its function as a biomolecule depot. Within the field of neural tissue engineering, these efforts are particularly evident in the creation of implantable materials or constructs for *ex vivo* expansion of stem and progenitor populations. These biomaterials are commonly made of natural polymers prevalent in the brain, such as hyaluronic acid, laminin, and heparan sulfate.^157–161^ Peptide sequences that enable matrix metalloproteinase (MMP)-degradability and cell adhesion via laminin and fibronectin recognition sites are often incorporated to facilitate neural cell migration alongside angiogenesis, as well as maintain proper cell physiology.^162,163^ While less abundant in the brain, collagen and gelatin have been used abundantly due to their proven effectiveness in promoting cell survival, growth, and migration in other engineered tissue systems, as well as their ease of injectability and polymerization.^164–168^ Subsequently, these materials are commonly loaded with growth factors to promote neural cell infiltration, proliferation, differentiation, survival, or angiogenesis. Examples include epidermal growth factor (EGF), vascular endothelial growth factor (VEGF), brain-derived neurotrophic factor (BDNF), glial cell-derived neurotrophic factor (GDNF), and SDF1α.^162,164,168–172^ Studies suggest that the use of laminin or GAGs synergizes with these growth factors by increasing receptor expression of implanted or infiltrating cells, and by sequestering the proteins for sustained delivery.^165,170^ Thus far, these materials have been effective in delivering, expanding, or recruiting neural populations for tissue repair post-stroke,^157,158,169^ improving the survival of sensitive populations such as dopaminergic neurons,^159,168,173^ and controlling cell function such as neural stem cell differentiation or astrocyte activation and quiescence.^162,163,167,174^ Beyond neural stem cell self-renewal and early differentiation, several long-term studies also demonstrate that biomaterials also support and hasten the maturation of functional neuronal subtypes compared to two-dimensional culture.^159,175–177^ Maturation is assessed by the emergence of mature neuronal markers via gene and protein expression, neurite extension and synapse formation, and electrophysiological assays such as whole-cell patch clamp that reveal that neurons are electrically active. Representative materials used to develop engineered brain-mimetic platforms are summarized in Table I.

**TABLE I.** Materials used in engineered brain-mimetic platforms. PEG: polyethylene glycol; PLLA: poly-L-lactic acid; PNIPAAm: poly(N-isopropylacrylamide).

Material	Application	References
Hyaluronic acid	Cell transplantation, growth factor delivery, disease model	134, 135, 158–162, 167, 169–172, 178, 213, 236
Laminin	Cell transplantation, disease model	170
Collagen	Cell transplantation, blood–brain barrier model, disease model, drug/growth factor delivery	149, 164, 167, 168, 178, 211, 213, 254
Gelatin	Disease model, cell recruitment, growth factor delivery, cell transplantation, *ex vivo* cell culture	71, 136, 160, 161, 165, 166, 234, 249
Alginate	Disease model	184
Matrigel	Organoid culture, disease model, *ex vivo* culture	167, 211, 238, 255, 264
Fibrin	Blood–brain barrier model	257, 258
Silk	Disease model, *ex vivo* cell culture	203, 205, 210
Chitosan	Disease model, *ex vivo* cell culture	174, 184
Chondroitin sulfate	Disease model	182
Heparin/heparan sulfate	Cell transplantation, disease model	158, 235
Xycoglucan/PLLA	Growth factor delivery, cell transplantation	173
PEG	*Ex vivo* cell culture, disease model	137, 138, 140, 163, 212, 235
PNIPAAm-PEG	Pluripotent stem cell differentiation	175
Elastin-like proteins	*Ex vivo* cell culture	141
Self-assembled peptides	*Ex vivo* cell culture, implantation, disease model	192–195
Decellularized extracellular matrix	*Ex vivo* cell culture, cell recruitment, pluripotent stem cell differentiation, disease model, growth factor delivery, implantation	196, 198, 199, 201, 202, 204, 265

The ability to create materials that mimic the composition of the extracellular matrix provides opportunities to create disease models for fundamental biology studies and drug screening. Many studies thus far have investigated the role of the extracellular matrix in GBM progression. Composite hydrogels derived from hyaluronic acid and collagen or gelatin have revealed that GBM cells preferentially spread and invade on collagen I or III instead of collagen IV, while increasing amounts of hyaluronic acid decreases cell spreading and migration.^178^ GBM phenotype is additionally dependent on the molecular weight of hyaluronic acid, with intermediate 60 KDa hyaluronic acid resulting in increased metabolic activity of GBM39 cells compared to 10 KDa and 500 KDa variants.^74^ Lower molecular weights of hyaluronic acid also support GBM invasion compared to 500 KDa polymers. Our lab has additionally shown that intermediate concentrations of hyaluronic acid (0.5 wt. %) in GelMA hydrogels significantly upregulates expression of genes related to GBM progression, such as *FN1*, *MMP9*, *VEGF*, and *HIF1A.*^179^ Recent efforts have focused on the role of hyaluronic acid in facilitating therapeutic resistance to receptor tyrosine kinase inhibitors (e.g., erlotinib).^70^ We have demonstrated that hyaluronic acid signaling through CD44 reduces erlotinib sensitivity by activating STAT3.^180^ A limitation of collagen and gelatin in these matrices, however, is the inability to precisely control the concentrations of degradation and adhesion sites. Alternative PEG-based materials have been utilized to independently modulate matrix components. Using this approach, Xiao *et al.* have identified that CD44 and integrin engagement through Arg-Gly-Asp (RGD) synergistically support therapeutic resistance to erlotinib and temozolomide.^137,181^ Materials containing GAGs beyond hyaluronic acid have also been developed, and studies show that sulfated GAGs such as chondroitin sulfate support GBM invasion better than hyaluronic acid due to sequestration of chemokines and activation of the associated cell receptors.^182^ Engineered materials also support the expansion of GBM stem cells, a rare but aggressive sub-population of the tumor believed to the responsible for recurrence and therapeutic resistance.^183,184^ Methods that preserve the self-renewal capacity and phenotype of these cells *ex vivo* is important for physiologically relevant benchtop studies and therapeutic discovery. While these strategies have yet been applied to develop models for neurological disease, we envision that adapting these platforms will enable significant insight into how extracellular matrix alterations contribute to degenerative disease onset and chronic progression.

While a vast majority of engineered biomaterials derive from natural biopolymers or synthetic materials, drawbacks include batch-to-batch variability, the use of potentially toxic reagents or toxic degradable byproducts, and the lack of control over material micro- and nanostructure. Inherently, higher-ordered structuring of proteins (i.e., secondary, tertiary, quaternary) is encoded in the amino acid sequence of the protein that directs resulting intermolecular interactions. These structures impact protein function and thereby influence cell behavior. In Alzheimer's disease, for example, amyloid plaques have a β-sheet secondary structure.^185^ Amyloid plaques are thought to contribute to disease progression in Alzheimer's and other neurological disorders.^186^ To design biomaterials containing these complex structural and biochemical cues, an area of ongoing innovation involves the development of biomaterials from self-assembled peptides.^187,188^ Custom peptides with user-encoded structural behavior can be created on a peptide synthesizer, and supramolecular interactions between peptides results in self-assembled hydrogels.^189^ In addition to mimicking the self-assembling nature of proteins, these materials demonstrate viscoelastic properties that can additionally be controlled by peptide design.^190,191^ In the realm of engineering brain microenvironments, these materials have been used to create hydrogels with synthetic amyloid-inspired fibrils to model Alzheimer's disease, expand and differentiate neural stem cells, and reduce reactive gliosis.^192–195^ Advancing the use of self-assembled peptide materials in the brain tissue engineering space will involve increased elucidation of assembled protein structures in the brain via chemical analysis and imaging tools, as well as collaborations with computational biologists and protein engineers to rationally design peptides to encode the proper structural and biochemical cues to mimic the brain microenvironment.

While increasingly sophisticated biomaterial fabrication and functionalization approaches offer the potential to recreate features of the native extracellular matrix, an orthogonal approach is the potential use of decellularized extracellular matrix. Decellularized extracellular matrix preserves the full complexity of the microenvironment in terms of its biochemical components, which is advantageous because cell behavior is likely a product of synergistic sensing of multiple matrix components and growth factors. While decellularized extracellular matrix can be used in a manner that additional preserves the *in vivo* structure of the brain tissue,^196^ many protocols solubilize the decellularized matrix and reconstitute the material as a hydrogel, which reduces structural resemblance to the original tissue.^197^ Sources of decellularized matrix include patient samples as well as animal-derived materials such as murine or porcine matrix. Decellularized matrix has been used as a regenerative tool, with injection or implantation of the material improving post-injury outcome and tissue repair in traumatic brain injury, stroke, and Parkinson's disease models.^196,198,199^ Decellularized matrix has also been used as a coating or scaffold to culture neurons or neural stem cells and direct the generation of neurons from induced pluripotent stem cells.^200–202^ Incorporating decellularized matrix into materials results in differentiated neural populations that are functionally mature; for example, Sood *et al.* show that neurons differentiated in silk scaffolds containing fetal decellularized matrix demonstrate increased spiking activity while differentiated astrocytes are star-shaped and phenotypically resting, not reactive.^203^ The use of decellularized matrix has also been used to culture GBM cells, in which tumor cells show varied morphologies and modes of migration (e.g., ameboid and mesenchymal) in contrast to those cultured in collagen gels, which favors a mesenchymal phenotype.^204,205^ Tumor cells cultured in decellularized matrix upregulate genes related to hyaluronic acid sensing and remodeling, as well as MMPs. Intriguingly, while migration decreases with HAS2 or MMP9 inhibition, cells switch morphology following inhibition, which may suggest reciprocal migratory mechanisms to evade therapy. Thus, the use of decellularized matrix recapitulates the heterogeneity of migratory phenotypes seen in native GBM, which allows for investigation of aggressive subpopulations and compensatory mechanisms that lead to treatment failure. While the use of decellularized matrix as a material shows exciting promise and underscores the importance of biochemical composition in these platforms, it is potentially limited by batch-to-batch variability, species mismatch, and supply. It is more sustainable to use the decellularized matrix as an inspiration with which to design engineered platforms. Here, techniques such as mass spectrometry can be used to characterize the decellularized matrix, and identified components can be incorporated into hydrogels derived from a synthetic template (e.g., PEG) or self-assembled peptides.^163^ In order to identify and optimize synergies between individual components, it will be useful to integrate microarray or combinatorial hydrogel platforms to perform high-throughput screenings of matrix components and their effect on cell behavior.^206,207^ Rational design-of-experiment strategies and regression modeling will be useful for navigating and optimizing the multidimensional biochemical landscape toward desired cellular outcomes. Such efforts will result in a user-defined decellularized matrix that is more reproducible and amenable to large-scale translation and clinical application.

### C. Cellular interactions

For platforms that serve as *in vitro* models to study stem cell biology and disease, or as delivery vehicles to introduce cellular payloads *in vivo*, it is important to identify suitable sources of cellular content to appropriately benchmark results. Cells encapsulated in engineered neural environments are typically derived from primary sources or from embryonic or induced pluripotent stem cell sources. Primary cells isolated from human or other mammalian sources are useful for fundamental biology studies and proof-of-concept experiments to demonstrate the effectiveness of potential tissue engineering strategies for transplantation.^70,134,157^ Given the potential difficulties of obtaining primary cells for transplantation, there is substantial interest in using cell populations derived from stem cell sources.^159,175^ Numerous protocols have been established for differentiating embryonic or pluripotent stem cells into neural or glial populations.^208,209^ Additionally, induced pluripotent or embryonic stem cell derived populations from diseased patients are being utilized to model various neurological disorders.^210,211^

Due to the diversity of cell types within the brain and the known contributions of cell–cell communication to overall tissue function, there has been recent interest in investigating heterotypic interactions between cohorts of dissimilar cells in engineered tissues. One approach mixes the different cell populations together in multi-culture. However, inherent challenges exist in tracing the behaviors of multiple cell populations and extracting cell-specific “-omics” (e.g., secretomics, proteomics, genomics, transcriptomics). In some cases, it may not be necessary to separate the responses of each cell type. Quantifying the population-level response may be sufficient in applications where the interest lies in assessing overall changes in the tissue, such as a global therapeutic response. For example, Schwartz *et al.* use RNA-seq to characterize transcriptomic changes of PEG hydrogels seeded with a mixed population of embryonic stem cell–derived neural progenitor cells, endothelial cells, mesenchymal cells, and microglia in response to a series to toxins to determine if the engineered cultures can be used to predict toxicology results.^212^ Shifts in the global transcriptome of the mixed populations are sufficient to train a machine learning model that correctly predicts the toxicity of 90% of test substances that are not used initially to train the model. For studies that intend to gain mechanistic insight, however, it is often essential to identify changes that arise from cell–cell interactions. For *in situ* observation of behaviors such as cell migration and proliferation, various fluorescent tags exist to label cell populations for short- and long-term periods. These include dyes and particles that are incubated with the cells right before use, or lentiviral constructs that are stably integrated into the genome through transduction. For instance, Herrera-Perez *et al.* label GBM cells with CellTracker Green before co-culturing them with astrocytes and endothelial progenitor cells in a collagen-hyaluronic acid hydrogel to track their migration.^213^ Increased migration is observed in the presence of astrocytes, while the effect of endothelial progenitor cells varies between the tumor cells tested. In another study, Xiao *et al.* transduce GBM cells with a lentivirus expressing luciferase in order to conduct bioluminescent imaging to monitor cell number in culture.^137^

Beyond tracking cells *in situ*, one might be interested in performing further analysis of cell state via the transcriptome, proteome, or other “-omes.” Obtaining-omics can be achieved through several methods with varying levels of specificity. One strategy is to compare the omics of the global population to controls derived from each individual cell type. For example, our research group has used RNA-seq to evaluate changes in the transcriptome that arise from interactions between GBM and vascular cells cultured in GelMA hydrogels.^214^ Here, we compare samples of RNA from hydrogels containing the GBM-vascular co-culture to mixed samples that we generated by combining RNA from vascular and GBM monocultures. Monoculture RNA is combined in accordance with the ratio of each cell type in the co-culture. Our results revealed that GBM–vascular interactions upregulate gene expression related to angiogenesis, extracellular matrix organization/remodeling, and invasion, while downregulating genes related to cell cycle and metabolism. Intriguingly, the analysis also reveals that GBM–vascular interactions dysregulate gene expression related to response to temozolomide, the standard-of-care chemotherapy for GBM; this signature is reinforced by functional dose-response assays that indicate that co-cultures are less responsive to temozolomide compared to the GBM monoculture. Such an experimental design is advantageous because cells do not undergo processing steps that change their phenotype before analysis; however, a limitation of this method is that phenotypic changes can only be attributed to the interactions between cell types, not to a specific cell type. In order to obtain cell type-specific information, the cell types of interest must first be isolated. For platforms in which the cell types are mixed together, the materials can be degraded to obtain a single-cell suspension,^215^ which is then processed using fluorescence-activated cell sorting (FACS) to obtain populations of interest via previously applied fluorescent tags or antibodies. If the engineered platform is designed to compartmentalize the different cell types, then the material itself can be dissected to obtain the different cell populations of interest. Alternatively, cells can be isolated by location within the material either through manual means or laser capture microdissection.^216^ Once isolated, the cells can be processed to obtain information at the transcript and protein level.

A drawback of any procedure that strives to isolate specific cell populations before analysis is that cell phenotype and viability can be affected during the isolation procedure. To minimize sample preparation before processing, ongoing innovations in next-generation sequencing and chemical biology offer tools to obtain cell-specific -omics without the need to physically separate the cells. Single-cell RNA-seq allows for transcriptomic profiling of individual cells from a heterogeneous suspension, in which cell types can be inferred from clustering techniques, identification of differentially expressed marker genes, and comparison to transcriptomic signatures of model cell populations from established datasets.^102,217,218^ Protein-level information can be obtained by using metabolic techniques such as stable isotope labeling, in which cells are fed with amino acids containing heavy and light isotopes that are subsequently integrated into peptides generated by the cells.^219,220^ Intracellular and secreted proteins can be resolved by cell type using mass spectrometry. A challenge in performing these -omics analyses is the need to distill large datasets into tangible mechanistic insights. Statistical techniques such as partial least squares regression (PLSR) can be used to produce multidimensional relationships between -omics products (e.g., secreted proteins, gene expression, etc.) and observed cell behavior (e.g., migration, proliferation, therapeutic response) in order to generate mechanistic hypotheses that link phenotype to genomic, transcriptomic, metabolomic, and proteomic outputs.^221–224^ Datasets can additionally be down-selected by benchmarking against “-omics” studies performed on primary patient specimens.^225^ Engineered platforms can subsequently be used to rapidly test hypotheses within physiologically relevant experimental conditions. For example, our lab recently employed a microfluidic invasion assay to perform high-throughput screening of vascular-secreted proteins for their potential contributions to GBM chemotactic invasion.^225^ Collaborations that combine expertise in chemical biology, bioinformatics, and molecular biology with biomaterials design will enable the integration of these various techniques and open the doors toward unprecedented insights in cell–cell communication in neurological disease and regeneration.

In applications such as regenerative medicine, delivering multiple cell types within a material may not be feasible or practical. As such, an alternative solution lies in engineering materials to contain the cues that cells use to communicate with each other. These cues may be secreted factors, juxtacrine-mediated, or manifested in extracellular matrix remodeling. Incorporation of secreted growth factors and extracellular matrix components has been discussed previously in this Perspective. Incorporation of cues arising from direct cell–cell contact has not been fully utilized in neural tissue engineering. Juxtacrine signaling includes cadherin-mediated and Notch pathways, with extensive literature implicating both in the regulation of stem cell behavior.^226–228^ Recapitulating cues that are normally presented by surrounding cells can be achieved by covalently tethering the cues to the matrix, or by encapsulating particles that are coated with the cues.^229,230^ This latter approach has been extensively explored in the immune engineering community to generate artificial antigen-presenting cells.^231^ We expect that combining paracrine, juxtacrine, and ECM-mediated signaling within engineered constructs will augment cell response for regenerative outcomes.

### D. Targeted control of oxygen tension

There are several methods to control oxygen tension for cell culture, including hypoxia chambers and chemical inducers, such as CoCl_2_.^124,232^ Our lab has cultured GBM cells encapsulated in gelatin hydrogels in a hypoxia chamber to observe the effect of 1% O_2_ on tumor cell behavior.^121^ This study revealed that reduced oxygen tension activates the “go-or-grow” phenotype, in which cell migration is enhanced while cell proliferation is inhibited. In another study, a blood–brain barrier model initially cultured in hypoxic conditions results in higher gene expression of cell adhesion and drug transport molecules, as well as transepithelial electrical resistance (TEER) values that are significantly higher and sustained over time than those measured after normoxic culture.^233^ An alternative strategy to generating a hypoxic environment is demonstrated by Sharon Gerecht's lab, in which gelatin modified with ferulic acid is enzymatically crosslinked using laccase and oxygen is consumed during the reaction.^124^ Continual consumption of oxygen during cell culture sustains hypoxic conditions within the hydrogel. We have adapted this platform to encapsulate neural stem cells in microfluidic devices to investigate the role of hypoxia and reactive oxygen species on neural stem cell differentiation and phenotype.^234^ Hypoxic culture conditions increase the reactivity of astrocytes as seen by glial fibrillary acidic protein (GFAP) staining, and both matrix stiffness and oxygen availability modulate the ability of neural stem cells to differentiate into neurons and astrocytes. Higher-stiffness materials promote neuronal differentiation, while softer materials promote astrocytic differentiation. Higher oxygen content also leads to increased spreading of astrocytes. Together, these capabilities highlight technological advancements that allow for precise control of oxygen levels in culture, thereby enabling studies that investigate how physiologically relevant fluctuations in oxygen availability direct brain development and disease progression.

### E. Neurodegenerative disorder models, brain organoids, and brain-on-chip

Besides GBM, the development of three-dimensional models of neurological diseases such as neurodegenerative disorders is still in its infancy. These models have significant potential for uncovering the specific mechanisms by which the brain microenvironment contributes to disease progression, which is currently unknown. Such knowledge will provide new therapeutic insights for stalling and potentially reversing progression for a series of diseases that are currently incurable. The development of neurodegenerative disease models is complemented by advances in cellular engineering and microfluidic technologies. In this section, we will discuss material-based microenvironments generated to model neurodegenerative diseases, opportunities to apply engineered materials to brain organoid development, as well as microfluidic strategies to mimic the brain (Fig. 3).

#### 1. General neurodegenerative disorder models

An essential benchmark for developing disease models is the ability to recapitulate hallmarks of disease phenotype or pathophysiological processes associated with disease progression. Being able to preserve the initial characteristics of the cell source is critical but can be difficult to achieve with fragile primary cell populations. Three-dimensional and organoid platforms have been used to preserve cell phenotype over long culture periods. Within the brain tissue engineering space, Rouleau *et al.* use silk scaffolds embedded with collagen to culture induced pluripotent stem cell-derived neurons and glia from healthy and Alzheimer's patients.^210^ Over a culture period of two years, these cultures remain viable, form neuronal networks, and Alzheimer's samples cultured for more than a year display increased markers of sporadic-Alzheimer's phenotype. Interestingly, these samples also show increased ROS activity, which may suggest a role for reactive oxygen species in more severe Alzheimer's phenotypes. Neuronal networks are also less active in Alzheimer's samples. Together, these results establish that *in vitro* microenvironments can maintain long-term cell stability. Another strategy used to study Alzheimer's disease involves treating cultures with amyloid beta (αβ) peptides. A study using this technique shows that αβ peptides alter the localization of pFAK in neurons, but the effect of this change is still unknown.^192^ Another study uses PEG-heparin gels to culture primary astrocytes and induced pluripotent stem-cell derived neurons, and cells pre-conditioned with αβ peptides show reduced neuronal network formation along with other hallmarks of Alzheimer's.^235^ IL-4 suppresses the phenotypic changes caused by αβ peptides by countering kynurenic acid production.

In addition to amyloid plaque accumulation, another microenvironmental change that occurs during Alzheimer's and other neurodegenerative disorders is the induction of inflammatory cues, which can also be simulated in *in vitro* models. To mimic multiple sclerosis, for example, Baisiwala *et al.* use a hyaluronic acid hydrogel to investigate neural stem cell differentiation to oligodendrocytes.^236^ Cultures in the presence of inflammatory cytokines reduces gene expression for myelin basic protein compared to controls, suggesting a detrimental effect of inflammation on re-myelination. These studies overall highlight the ability of engineered materials to mimic aspects of neurodegenerative disorders and provide mechanistic insights that can potentially be translated into therapies.

#### 2. Brain organoids

Organoids are broadly defined as three-dimensional cultures of primary or stem cells that self-organize to recapitulate the morphology and function of target tissues.^237^ Advancements during the past decade have led to the establishment of brain organoids from induced pluripotent or embryonic stem cells that mimic some of the cellular diversity and regional specification seen in the brain.^238,239^ Protocols for organoid development typically involve culturing the stem cells in chemically defined media that promotes specification to the neural lineage and directs differentiation.^240^ Media can be supplemented with various inhibitors or growth factors to mimic the cascade of morphogens present during development. Limitations of current organoid protocols include batch-to-batch variability, lack of spatial morphogen patterning, core necrosis due to lack of vascularization, and organoid immaturity. Given that tissue development is partially influenced by the extracellular microenvironment, recent efforts to integrate engineered biomaterial microenvironments with organoid cultures offer the exciting opportunity to enhance the homogeneity or specificity of resulting brain organoids as well as the opportunity to formalize high-throughput culture tools necessary to use organoid technologies in drug development.

Because Matrigel supports epithelium growth and function, it is commonly used in brain organoid protocols to generate neuroepithelium.^240,241^ However, Matrigel suffers from batch-to-batch variability that can eventually manifest in reproducibility issues between cultured organoids.^242^ In recent years, the biomaterials community has explored the use of synthetic, PEG-based hydrogels to expand intestinal organoids, with outcomes that are equivalent to, if not better than, standard Matrigel protocols.^243^ Therefore, it is interesting to consider the use of brain-mimetic matrices to develop brain organoids. Not only would this improve reproducibility, but mechanical and biochemical properties are additional and unexplored variables with which to optimize organoid formation. Engineered approaches also provide an avenue to spatiotemporally pattern morphogens to guide regional specification. Approaches to pattern morphogens include the use of photolabile linkers to tether and release proteins from the material on demand, or the use of microfluidic strategies to establish soluble and tethered gradients of protein.^244–247^ While embryonic brain development begins in the absence of vasculature, infiltrating vascularization is eventually necessary to supply nutrients and oxygen for tissue growth and to provide niche cues for neural stem cells.^248^ Brain organoids can be cultured with endothelial and perivascular stromal cells within biomaterials to produce capillary networks that contact and penetrate the organoid.^249,250^ Currently, vascularized organoids have to be implanted into *in vivo* models in order to obtain perfused vasculature.^251^ Alternatively, 3D printing strategies can be used to embed organoids in networks of hollow channels that can be lined with endothelial cells and perfused *in vitro.*^252,253^ Vascularizing brain organoids will advance their developmental maturity beyond what is currently possible, therefore expanding the ability to study a fuller timescale of human brain development.

#### 3. Brain-on-a-chip platforms

The incorporation of microfluidic technologies into engineered platforms enables exploration of additional variables such as interstitial and intraluminal flow. An evolving area of focus in the research community involves modeling the blood–brain barrier in order to understand drug transport phenomena, therapeutic angiogenesis, and the implications of vascular breakdown on disease. Several methods for mimicking the blood–brain barrier exist. In one approach, a hydrogel can be cast around a cylindrical object, which is then removed to leave behind a hollow channel that can be lined with endothelial cells. To fully recapitulate the blood–brain barrier using this approach, Herland *et al.* line a collagen channel with pericytes and endothelial cells, while astrocytes are dispersed throughout the hydrogel.^254^ Astrocytes and pericytes are essential for reducing permeability values to levels on par with other blood–brain barrier models. Another technique involves lining microfluidic device channels or membranes with endothelial cells, such that the fluidic channel itself serves as a vascular lumen. This setup has been used to investigate blood–brain barrier dysfunction in Alzheimer's disease by co-culturing endothelial cells with normal or familial Alzheimer's disease (FAD)-mutated neural progenitor cells.^255^ Here, the presence of mutated cells increases barrier permeability along with reactive oxygen species, MMP2, and IFNγ. Barrier dysfunction also leads to neural death after thrombin is introduced into the platform. However, barrier dysfunction can be reversed by removing amyloid plaques or by applying therapeutics that promote barrier function. This suggests that the platform can be used to screen novel agents of barrier repair and further reinforces a need to mechanistically link the presence of amyloid plaques to pathophysiological phenotype. A final approach involves the generation of microvascular networks within microfluidic hydrogels, which can be perfused through flanking fluidic channels.^256,257^ Such an approach can be used to investigate nanoparticle penetration as well as intravasation and extravasation events during cancer metastasis.^258–260^ Microfluidic devices can be fluidically linked to create integrated tissue or organ systems;^261^ for example, Maoz *et al.* connect two vascular chips with a brain chip to mimic therapeutic delivery into and out of the brain from vasculature.^262^ This strategy recapitulates physiological-relevant pharmacokinetics as drug compounds travel through circulation, across vascular barriers, and through tissue. In addition to drug studies, this approach allows for investigation into paracrine signaling between tissues or organs. For example, Maoz *et al.* demonstrate that vascular signaling increases metabolic output by astrocytes and neurons and the synthesis of the neurotransmitter gamma-aminobutyric acid (GABA).^262^ Overall, coupling of microfluidic devices presents a promising strategy to investigate systemic toxicity and signaling across tissues and organs. Intriguing applications include modeling the gut-brain axis, the metastatic cascade from a primary tumor to the brain, or the infiltration of circulating immune cells across a compromised brain microvasculature.

A second advantage of on-chip platforms is the ability to control the spatial organization of the engineered tissue. Function often follows form for biological tissues; for instance, proper patterning of vasculature is necessary to deliver nutrients and oxygens throughout tissues.^263^ User-defined compartmentalization of different cell types within an engineered construct is one method to control spatial organization in engineered systems. Such spatial organization can be programed through the design of microfluidic devices. To create an amyotrophic lateral sclerosis (ALS) model, Osaki *et al.* developed a device in which myoblasts are seeded in a compartment with two pillars to form muscle bundles, while motor neuron spheroids are injected into an adjacent compartment.^211^ After fourteen days, neurons extend toward and contact the muscle bundles to form a neuromuscular unit that undergoes muscle contractions. To stimulate ALS conditions, cultures can be spiked with glutamate, or optogenetics can be used to stimulate motor neurons derived from an ALS patient. With both methods, muscle contractions become weaker and less frequent, and the neuromuscular unit regresses. Related efforts have also employed microfluidics to pattern neurons, astrocytes, and microglia to develop an Alzheimer's model.^264^ Neurons and astrocytes are cultured in the center of the platform, while microglia are placed in a concentric region. Thin pathways connect the core and outer compartments, such that microglia will only migrate into the core in response to soluble factors. To model Alzheimer's, neurons and astrocytes are differentiated from progenitor cells overexpressing amyloid beta precursor protein. The model shows increased secretion of amyloid beta peptides and phosphorylated-tau, along with inflammatory proteins that recruit microglia toward the central compartment. Recruited microglia increase the pro-inflammatory profile of the secretome and negatively impact neuronal phenotype through IFNγ and TLR4.

3D printing is another strategy with which to generate spatially organized tissue models. In one example, a GBM-on-chip is created by printing concentric circles of endothelial and tumor cells in bioink derived from decellularized matrix.^265^ Oxygen permeability is restricted to the radial direction, creating a zone of hypoxia in the core of the design. Only in the presence of an oxygen gradient and separated cell types does the platform recapitulate pseudopalisade formation and retains a fraction of SOX2+ stem-like tumor cells, which are both hallmarks of GBM progression. Beyond the ability to spatially position multiple cell types,^266^ 3D printing also enables spatial patterning of biophysical properties. For example, Tang *et al.* develop a GBM model consisting of a core of tumor cells surrounded by an acellular matrix region with either low or high stiffness to mimic the variations in matrix stiffness found in GBM tissue.^267^ Tumor cells printed alongside stiff matrices display upregulated gene expression related to hypoxia and tumorigenicity and are more similar to primary GBM specimens compared to tumor cells printed with soft matrices. Furthermore, the use of soft or stiff matrices leads to the emergence of divergent tumor subtypes, with the stiff model promoting mesenchymal and proneural subtypes while the soft model supports the classical subtype. These studies highlight the importance of recapitulating spatial heterogeneity in engineered microenvironments as a means to better mimic *in vivo* behavior.

Taken together, chip technologies offer the opportunity to enhance the physiological relevance of engineered models by enabling spatial patterning, fluid flow, and modular development of integrated tissue mimics. Most excitingly, these innovations are scalable and can be designed to culture small numbers of cells, which is valuable for high-throughput experimentation and screening and handling rare populations of primary or patient-derived cells.

## IV. PERSPECTIVE AND CONCLUSIONS

In this Perspective, we have discussed the biophysical, biochemical, cellular, and metabolic attributes of the native brain tissue. These parameters often serve as inspiration for engineered systems to enhance neural regeneration, model disease, screen therapeutics, and understand stem cell biology. We highlight state-of-the-art tools and strategies to create highly defined and physiologically relevant materials, identify areas for ongoing innovation, and present opportunities for collaborations beyond the tissue engineering community to accelerate the widespread translation of these materials in clinics and research laboratories. Here, we will conclude with a few broad considerations for the field to consider moving forward.

### A. Personalized engineered microenvironments

Brain tissue mechanical properties vary based on spatial location within the tissue, sex, and age. A one-size-fits-all approach is likely not appropriate for designing implantable materials for regeneration, and different patients may require customization to achieve ideal outcomes. *In vitro* studies that utilize cells sourced from different sexes and ages will give insight into the extent of customization needed to achieve optimized cell behavior. Correspondingly, engineered materials as culture platforms should incorporate the necessary extracellular differences observed with sex and age to generate environments that are physiologically relevant to a diverse range of patients. Such materials can then be used to gain insight into how sex and age influence differences in disease onset and progression across the patient population. However, progress in this space will require an entirely new approach in bioengineering that centers on the idea of contextual complexity. While our field has focused on knowing more (e.g., via miniaturization, parallelization, quantitative biology) over the last twenty years, future progress requires understanding complexity in context, using lenses of variation and heterogeneity as well as acknowledging the limits of what can be measured within complex biological and social environments. As a field, we must continue to broaden participation in collaborative teams and include diverse expertise in critical studies to enable a new generation of reflexive bioengineering initiatives.

### B. Dynamic materials for dynamic microenvironments

Tissue repair, regeneration, and disease are temporally dynamic events accompanied by changes in extracellular matrix properties and cellular behavior. Assessing how shifts in the extracellular environment affect cell behavior, and vice versa, is essential for understanding how regenerative events and disease progress. Identifying whether changes lead to positive or negative outcomes informs therapeutic strategies that can guide the tissue toward a repaired state. For regenerative materials, knowledge that different material stiffness and degradative regimes control neural stem cell expansion and differentiation motivates the need to design materials with dynamic mechanical properties to control timescales for cell infiltration, proliferation, and differentiation into the diversity of cell types needed to restore tissue function. Several strategies exist to design dynamic engineered materials that mimic the spatiotemporal variations in native tissue. For example, materials can be designed to soften or stiffen based on user-controlled cues such as light or intelligent design of degradable crosslinks.^144,268,269^ Proteins can be added or removed from materials by using orthogonal and reversible tethering strategies.^244,270^ Cells can also be controlled using light- or chemically inducible gene expression;^271–273^ this is useful for turning on and off genes to understand how genetic alterations impact the surrounding microenvironment. Incorporating these strategies when engineering materials will be particularly useful for understanding how changes in the extracellular matrix during neurodegenerative disease leads to chronic conditions.

### C. Experimental and computational tools to navigate complexity

It is evident that the brain microenvironment is complex and features overlapping biophysical, biochemical, metabolic, and cellular cues that vary spatially and temporally. Cell phenotype arises from synergistic sensing and processing of these overlapping cues, and therefore it is likely that the design of both regenerative and stem cell platforms and disease models will require careful optimization of multiple design variables. It will be beneficial to adapt technologies that enable efficient and rational exploration of these multivariate design spaces. For example, employing design of experiments and regression analyses will facilitate the development of studies that logically explore and elucidate the relationship between combinatorial microenvironmental cues and cell behavior.^162,221,274^ Employing these approaches first enables a comprehensive exploration of the microenvironmental space, and then an avenue to distill large datasets of experimental results into a tangible model that identifies the key microenvironmental cues responsible for a given phenotype. Collaborations that combine engineering, statistical, and bioinformatics expertise will be beneficial for merging these statistical tools with tissue engineering paradigms. Additionally, the tissue engineering field has developed multiple high-throughput platforms (e.g., microarrays, gradient materials) to generate large libraries of combinatorial microenvironments.^207,275,276^ Employing these combinatorial approaches will enable efficient screening of cell phenotypes that arise from different synergies between microenvironmental cues.

Overall, rapid advancements in engineering precise and physiologically mimetic brain microenvironments during the past decade have brought us closer to strategies to treat and repair neurological disorders and injuries. While several of these diseases are still considered to be incurable, we believe that increased understanding of microenvironmental barriers to recovery will provide new therapeutic targets, and tissue engineering strategies to deliver and sustain therapeutic payloads will facilitate tissue repair and bolster patient outcomes. Engineered brain microenvironments have great potential as tools at the benchtop and the clinic and will contribute significantly to neuroscience and neurology for the decades to come.

## Data Availability

Data sharing is not applicable to this article as no new data were created or analyzed in this study.
